# Peripheral vision as a key factor for decision-making in soccer

**DOI:** 10.3389/fpsyg.2026.1907506

**Published:** 2026-09-28

**Authors:** Lovro Ivosevic, Tino Stöckel

**Affiliations:** Sport & Exercise Psychology Unit, Department of Sport Science, University of Rostock, Rostock, Germany

**Keywords:** decision accuracy, decision time, peripheral vision, soccer, speed-accuracy trade-off

## Abstract

Peripheral vision is critical for processing dynamic visual information in team sports such as soccer; however, yet little is known about how individual differences in peripheral visual field extent relate to decision-making efficiency under time pressure. This study investigated whether peripheral visual field size is associated with decision-making performance in soccer players while controlling for competitive level. Forty male soccer players (age: 21.65 ± 3.92 years) completed a peripheral vision assessment using a BlazePod system positioned at eccentricities ranging from 60° to 100°, while maintaining central fixation under eye-tracking control. Decision-making performance was assessed using 22 video-based soccer scenarios (presented twice) that required rapid pass/no-pass decisions. A composite performance ratio integrating decision accuracy and decision time served as the primary outcome. Partial correlation analyses, controlling for playing level, revealed a significant positive association between peripheral visual field extent and the performance ratio. This relationship was more strongly reflected in faster decision times, whereas the association with decision accuracy was weaker. These findings suggest that a larger peripheral visual field is associated with more efficient decision-making in soccer, largely due to reduced response times rather than improved accuracy. Peripheral visual capacity may therefore contribute uniquely to perceptual-cognitive performance beyond competitive level and could represent a potential target for future training interventions in time-constrained sport settings.

## Introduction

Peripheral vision – defined as the ability to process visual information outside the foveal (central) line of sight (Vater et al., 2019) – is a fundamental perceptual skill in many every day and sport-specific contexts. Its relevance in elite sport was recently illustrated during the UEFA EURO 2024 championship, when Kylian Mbappé was required to wear a protective mask following a nasal fracture. The mask was reported to restrict his peripheral vision, prompting discussions among coaches and sport psychologists regarding the impact of such a restriction on perceptual processing and decision-making performance on the pitch. In match situations such as attacking down the wing, players like Mbappé typically rely on peripheral vision to monitor teammates’ off-the-ball runs into the penalty area as well as approaching defenders, without the need to shift gaze away from the ball. Reduced peripheral visual capacity may therefore delay information pickup and impair rapid decision making, potentially leading to missed passing opportunities (Klostermann et al., 2020).

The importance of peripheral vision extends far beyond elite soccer. In daily life, peripheral vision is essential for detecting obstacles while navigating crowded environments or for monitoring surrounding traffic, such as noticing a vehicle entering an adjacent lane while driving (Wu et al., 2025). In sports, however, the demands placed on peripheral vision are particularly pronounced. In team sports such as soccer, handball, and basketball athletes must continuously process multiple players and dynamic spatial relationships while simultaneously maintaining attention on the ball or a direct opponent (Vater et al., 2019). Empirical evidence from mobile eye-tracking research further indicates that soccer players rely heavily on peripheral vision during match play, as gaze fixations alone cannot fully account for the rapid acquisition and integration of visual information required for split-second decision-making (Aksum et al., 2021). Peripheral vision enables rapid extraction of spatial information without the need for frequent gaze shifts, thereby reducing processing delays in dynamic environments (Stankov et al., 2021). This continuous stream of visual information may facilitate more efficient decision-making by supporting the integration of multiple moving elements under time constraints. In dynamic settings, monitoring targets through peripheral vision may even be optimal, given the temporal costs associated with saccadic eye movements (Vater et al., 2016).

Recent empirical work has begun to clarify how peripheral vision operates in dynamic sport environments. Vater et al. (2026) examined peripheral vision in a virtual-reality soccer counterattack task and found that increasing viewing angles significantly impaired both detection accuracy and response time, with reduced performance observed at 60° eccentricity compared to 20° or 40°. Interestingly, although participants appeared to rely more heavily on peripheral vision at greater eccentricities, performance still declined, suggesting that initiating a saccadic eye movement toward peripheral stimuli may, in some situations, be more effective than relying on peripheral processing alone (Vater et al., 2016). Complementing these findings, DeCouto et al. (2023) demonstrated that peripheral vision enables athletes to integrate multiple visual cues across different regions of the visual field, thereby supporting performance in complex, dynamic situations. From a neurocognitive perspective, Piras (2025) distinguished between top-down and bottom-up attentional processes, suggesting that peripheral information processing is influenced by both task demands and stimulus saliency. Moreover, early evidence by Stark and Bridgeman (1983) and Mizusawa et al. (1983) showed that athletes exhibit superior peripheral motion perception compared with non-athletes, indicating that sport-specific experience may enhance peripheral visual capabilities. More broadly, reviews on perceptual-cognitive expertise indicate that successful decision-making in team sports depends on the effective integration of central and peripheral visual information, efficient gaze strategies, and anticipation of opponents’ actions (Mann et al., 2007; Vickers, 2007; Williams and Ford, 2008).

The concept of “visual pivots” has been proposed to describe how skilled athletes anchor their gaze on a central reference point (e.g., the player in possession) while monitoring surrounding information through peripheral vision (Vater et al., 2019). This strategy is thought to reduce the need for frequent saccadic eye movements, thereby minimizing periods of visual suppression during gaze shifts, which is particularly advantageous under high temporal constraints (Vater et al., 2019). In soccer, for example, highly skilled players have been shown to anchor their gaze on the player in possession of the ball while simultaneously using peripheral vision to track the movements of surrounding players in small-sided (3 vs. 3) game situations (Vater et al., 2019).

While previous research has substantially advanced our understanding of peripheral vision in sport, research has predominantly focused on basic perceptual capabilities or group-level comparisons between expert and novice athletes (e.g., Panchuk and Maloney, 2022; Savelsbergh et al., 2005; Williams et al., 2011). Consequently, relatively little is known about how individual differences in peripheral visual performance relate to decision-making efficiency in sports. Furthermore, it remains unclear whether peripheral vision contributes uniquely to decision-making performance beyond the influence of a player’s competitive level. Evidence from other domains suggests that a larger peripheral visual field is associated with superior performance in decision-making and visual processing tasks. For example, peripheral acuity has been linked to performance in artificial target-search tasks (Leachtenauer, 1978), and the Useful Field of View (UFOV) has been shown to predict driving performance in older adults (Clay et al., 2005). Drawing on these findings, we hypothesized that a larger peripheral visual field would be associated with greater decision-making efficiency in soccer players. Accordingly, the present study investigated whether individual differences in peripheral visual field extent are associated with decision-making efficiency under time pressure. In addition, we examined whether this relationship persists after accounting for players’ competitive level.

## 2 Methods and materials

### 2.1 Participants

A total of 46 participants were recruited for the study. Six participants were excluded following screening due to previously undiagnosed red-green color deficiency. The final sample comprised forty male soccer players (age: 21.65 ± 3.92 years; playing experience: 14.75 ± 4.83 years). Participants competed at levels ranging from the fourth to the ninth tier of the German league system. The final sample comprised forty male soccer players (age: 21.65 ± 3.92 years; playing experience: 14.75 ± 4.83 years). Participants competed at levels ranging from the 4th to the 9th tier of the German league system. Specifically, one participant competed in the 4th tier (*Regionalliga*), seventeen in the 5th tier (*Oberliga*), seven in the 6th tier (*Verbandsliga*), eight in the 7th tier (*Landesliga*), five in the 8th tier (*Bezirksliga*), and two in the 9th tier (*Kreisliga*). Youth league classifications were converted to equivalent senior league tiers to facilitate comparison across participants (Wozniak, 2019). Participants were recruited through a public call and a local youth academy. Inclusion criteria comprised male participants aged 16–35 years who were actively participating in competitive soccer, irrespective of performance level. Exclusion criteria included the presence of any diagnosed visual impairment and color vision deficiencies. The study was approved by the local institutional review board at the University of Rostock (A2025-0135) and was conducted in accordance with the Declaration of Helsinki. All participants provided written informed consent prior to participation and were informed of their right to withdraw from the study at any time. Participants did not receive compensation. An *a priori* power analysis was conducted using G*Power (Erdfelder et al., 1996). Assuming a large effect size (r = 0.5; e.g., Leachtenauer, 1978; Clay et al., 2005), a one-tailed test, an alpha level of α = 0.05, and desired power of 1-β = 0.90, the analysis indicated a required minimum sample size of N = 31 participants to detect the expected effect.

### 2.2 Measures and experimental procedure

All testing sessions were conducted individually in a controlled laboratory setting and lasted approximately 45–60 min. Upon arrival, participants received standardized instructions and completed a brief familiarization with the BlazePods to ensure all colors were properly recognized. The experimental procedure consisted of two parts administered in a fixed order: (1) peripheral vision assessment, followed by (2) decision-making assessment. Participants were instructed to respond as quickly and accurately as possible in all tasks.

Peripheral vision was assessed using the BlazePod system (BlazePod Inc., Miami, FL, USA), which was controlled via a dedicated mobile application (BlazePod, 2018). The BlazePod system has demonstrated construct validity as a reliable tool for assessing visual reaction time and peripheral detection. de-Oliveira et al. (2021) reported moderate to excellent test–retest reliability (ICC = 0.82 for response time), with acceptable measurement error for practical applications. Furthermore, Piva et al. (2025) investigated the effects of an 8-week soccer-specific vision training program using the BlazePod system in young soccer players and reported significant improvements in visuomotor reaction time and saccadic precision, further supporting its utility in sport-specific settings. Collectively, these findings support the validity of the BlazePod system as a tool for assessing visuomotor performance. Testing was conducted in a darkened room under standardized lighting conditions. Ten circular BlazePods were arranged in a semicircular configuration on tables 45 cm high, positioned at visual angles of 60°, 70°, 80°, 90°, and 100° relative to a central fixation point (i.e., participants’ sitting position, see Figure 1). The distance between the participant and the pods was 1.5 m, with the pods a few centimeters below eye-level. The protocol comprised two trials of 4 min each, separated by 1-min rest intervals. During each trial, the BlazePods illuminated in a randomized sequence for 0.5 s at interstimulus intervals ranging from 2 to 4 s, displaying one of four colors (blue, pink, red, or green). The protocol was standardized such that each BlazePod at every eccentricity level (60°, 70°, 80°, 90°, 100°) was illuminated a minimum of 10 times across the trials, ensuring equal presentation frequency across all visual angles. Participants were instructed to maintain fixation on a centrally positioned cross at eye-level ahead of them and to respond to peripheral stimuli as quickly as possible without moving their gaze. Eye movements were continuously monitored using an eye-tracking system (Be Gaze SMI 3.7.60 software, SensoMotoric Instruments Inc., Boston, USA; sampling rate: 250 Hz), which was calibrated using a standard 9-point calibration procedure prior to testing. The primary outcome measure for the peripheral vision task was the visual angle at which participants reliably (i.e., at least 50% correct responses) detected a peripheral stimulus. The visual angle was estimated using linear interpolation between two adjacent measurement points, defined by the 50% correct response threshold. The threshold visual angle was estimated using linear interpolation, defined as:

**FIGURE 1:**
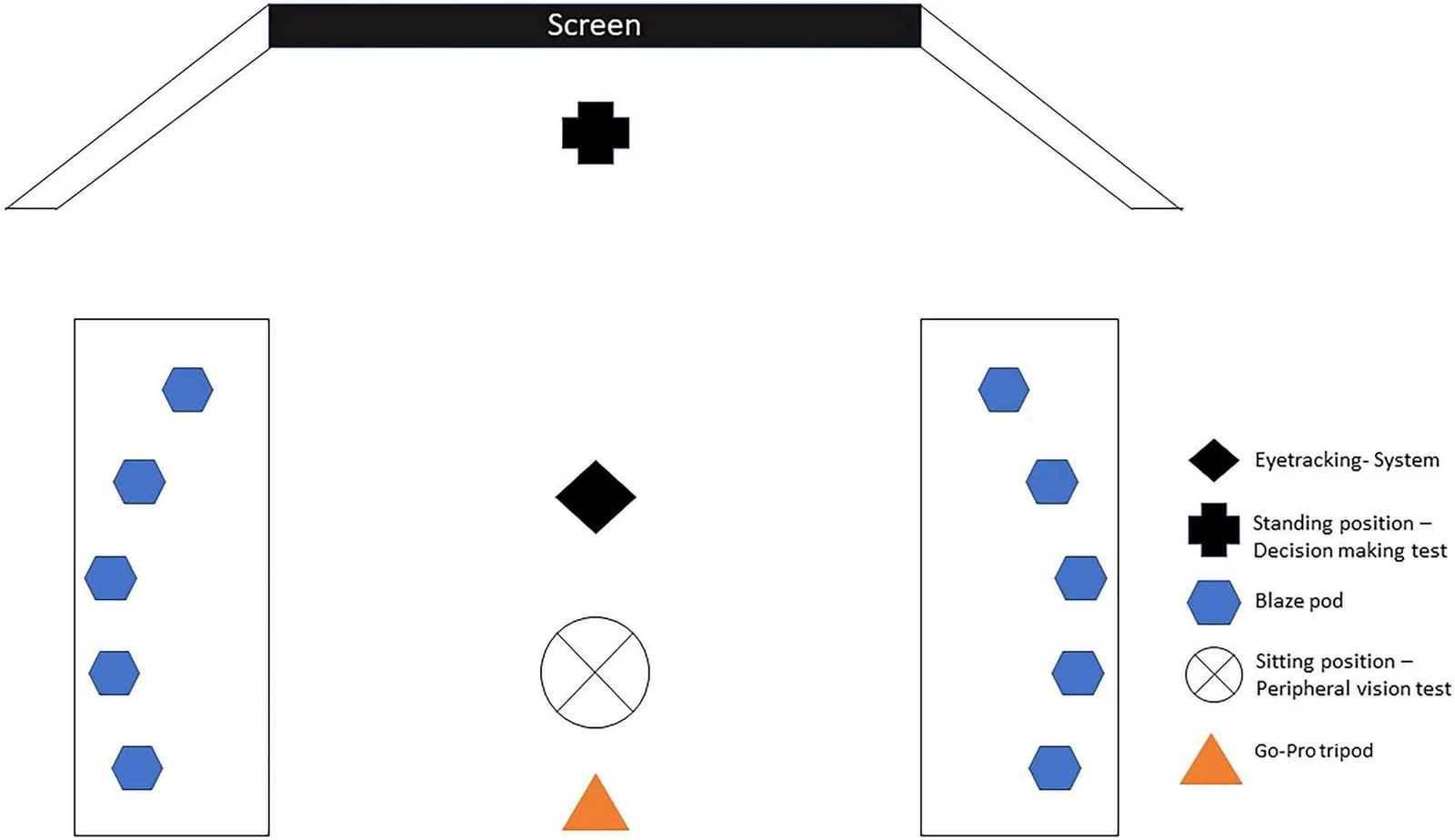
Top view of the experimental setup.


$${\text{x}}_{p}={\text{x}}_{1}+\frac{\text{H}-{\text{H}}_{1}}{{\text{H}}_{2}-{\text{H}}_{1}}*\left({\text{x}}_{2}-{\text{x}}_{1}\right)$$

where *H* denotes the target performance level (i.e., hit rate of 50%), *x*_1_ and *x*_2_ are adjacent measurement points and *H*_1_ and *H*_2_ the performance level (i.e., hit rate) at *x*_1_ and *x*_2_ respectively. The 50% detection threshold is a standard, validated, and physiologically grounded measure in visual perception research and is defined as the minimum stimulus intensity required for detection in 50% of trials (Spielman et al., 2020, Chapter 5.1). Furthermore, the use of a 50% detection criterion is consistent with established psychophysical procedures employed in clinical perimetry, including the Humphrey Field Analyzer (HFA; Heijl et al., 1989), thereby supporting methodological comparability. The HFA assesses visual field sensitivity by presenting light stimuli of varying intensities at predefined locations within a white hemispherical bowl while participants maintain fixation on a central target and stimulus detection via a response button. According to standard clinical references, the normal monocular visual field extends approximately 90 to 100 degrees laterally from the point of fixation (Walker et al., 1990). In the present study, the mean lateral visual field measured 94 degrees on both sides, demonstrating comparability with the visual field covered by the BlazePod measuring system. All trials were recorded using a GoPro HERO7 Black camera (GoPro Inc., San Mateo, CA, USA) for subsequent analyses (Figure 1).

Decision-making performance was assessed using 22 video sequences with each sequence presented twice to the participants. The scenes were recorded from the perspective of a ball-carrying player positioned centrally a few meters within his own half and facing the opposing goal. The scenes were filmed at a local youth soccer academy training facility using a GoPro HERO7 camera mounted at a height of 175 cm to approximate players’ eye-level. In each sequence, one attacking player was positioned on each wing and ran from the midfield line toward the opponent’s goal while clearly anticipating a pass or not. Across sequences, the wing players differed in their tactical availability, with one or both players either closely marked by an opponent or left unmarked. The specific game scenario (two wingers, two defenders, and one goal) was selected to systematically represent all possible passing options to the left and right wings while maintaining a controlled task structure. The first level of complexity included one attacker on each side without defensive pressure. In the second level, one defender trailed one of the attackers, whereas the attacker on the opposite side remained unmarked. In the third level, both attackers were marked by a defender. In the fourth and final level, both attackers additionally used hand signals to indicate that they were available as legitimate passing options, thereby further increasing the perceptual and decision-making demands of the task. To establish the correct response for each sequence, three expert raters (two youth soccer coaches and one former professional player) independently evaluated the scenarios and determined whether the optimal decision was a pass to the left wing, a pass to the right wing, a pass to either wing, or no pass.

The scenes were displayed on a 98-inch monitor (see Figure 1) in a randomized order using BeGaze SMI 3.7.60 (SensoMotoric Instruments Inc., Boston, USA), and viewing distance (80 cm) as well as eye height were standardized to the height of the goal crossbar using a reference still image and a marked floor position. Prior to data collection, we verified that the entire display was within the participants’ visual field by measuring the horizontal and vertical visual angles from the standardized viewing distance of 80 cm. Based on these measurements, the wingers in the video stimuli were positioned at an estimated eccentricity of approximately 35°–45° relative to the center of the screen, where the ball carrier was located. Thus, all task-relevant visual information fell within the estimated peripheral visual field. This eccentricity range is consistent with previous research by Hüttermann and colleagues on the spatial distribution of visual attention and decision-making in sport (Hüttermann and Memmert, 2017). Participants first completed a practice trial, after which all 44 experimental trials were presented in a randomized order. No auditory information was provided. Participants were asked to verbally indicate whether they would pass the ball to the right, to the left, or whether they would not pass the ball at all as fast and as accurate as possible. Participants were instructed to make a passing decision based on the available passing options. Alternative actions (e.g., shooting, dribbling, or delaying play) were not considered, as the task was designed exclusively to assess passing decision-making. Response time was defined as the interval between the onset of the video stimulus and the initiation of the participant’s verbal response. Verbal responses were recorded using the integrated audio-recording functionality of the BeGaze system (SensoMotoric Instruments), which synchronizes audio recordings with the video stimulus presentation and enables millisecond-level determination of response timing. Response time and decision accuracy served as the primary dependent variables and were analyzed separately. In addition, a composite performance score was calculated for each participant as the ratio of accuracy to mean decision time. This performance ratio was included as an integrated measure of decision efficiency, combining the two theoretically relevant aspects of performance: speed and accuracy. Such integrated speed–accuracy measures have been widely used in cognitive research when both performance components contribute to the construct of interest (Vandierendonck, 2017). Specifically, the ratio of correct responses to response time can be interpreted as the rate at which accurate decisions are achieved, providing complementary information about overall performance efficiency that may not be fully captured by accuracy or response time alone (Urry et al., 2015).

### 2.3 Data analysis

Pearson correlation analyses were performed using IBM SPSS Statistics (version 31.0.0.0.; IBM Corp., Armonk, NY, USA) to examine the relationships between peripheral visual field extent and decision-making performance in soccer. Decision-making performance was quantified using decision quality (accuracy based on expert ratings), decision time (time required for participants to verbally announce their decision), and an integrated performance ratio (accuracy divided by decision time), which captures decision efficiency by considering both speed and accuracy. As participants competed across a wide range of competitive levels, playing level was included as a categorical covariate in the regression analyses, with the league tiers (4th–9th) represented using dummy coding and the 4th tier serving as the reference category. Correlation coefficients were interpreted according to Cohen’s (1988) conventions: r = 0.10, 0.30, and 0.50 representing small, medium, and large effects, respectively. Because the hypotheses were directional, one-tailed tests were used for all correlation and regression analyses. Statistical significance was set at 0.05. Prior to conducting the correlation and regression analyses, the relevant assumptions were examined. Linearity was assessed through visual inspection of scatterplots, normality was evaluated using the Shapiro–Wilk test, and homoscedasticity was assessed through inspection of residual plots. No substantial violations were identified (all Shapiro–Wilk tests: *p* > 0.05), and no influential observations were detected (Cook’s distance < 1).

In addition to correlation analyses, ordinary least squares (OLS) regression models were estimated for all reported relationships. Multiple linear regression analyses were conducted using JASP (version 0.96) to examine the association between peripheral visual field extent (independent variable) and the performance ratio (dependent variable), while accounting for playing level as a categorical covariate using the dummy-coded league tiers described above. Simple linear regression models were conducted to examine the relationships between peripheral visual field extent (independent variable) and decision accuracy and decision time (dependent variables). All regression coefficients were estimated using the ordinary least squares method.

## 3 Results

Descriptive statistics for all primary variables were calculated. Peripheral visual field extent ranged from 163.56° to 205.90° (M = 188.17°, SD = 9.96°). Decision accuracy ranged from 0.455 to 0.773 (M = 0.627, SD = 0.067), and decision time ranged from 3.20 s to 10.70 s (M = 6.48 s, SD = 2.38 s). The performance ratio (accuracy divided by mean decision time) ranged from 0.060 to 0.186 (M = 0.109, SD = 0.037). A multiple linear regression was conducted to examine the association between peripheral vision and performance ratio while accounting for playing level as a categorical variable using dummy coding. The overall model was significant, F(6, 33) = 4.407, *p* = 0.002, R^2^ = 0.445, adjusted R^2^ = 0.344. Peripheral vision remained significantly positively associated with performance ratio after accounting for playing level (B = 0.002, SE = 0.0006, β = 0.574, t(33) = 3.75, *p* < 0.001), indicating that greater peripheral visual field extent was associated with better decision-making performance. Adding playing level to the model explained an additional 16.5% of the variance in performance ratio, although this increase was not statistically significant (ΔR^2^ = 0.165, *p* = 0.110) (Figure 2).

**FIGURE 2:**
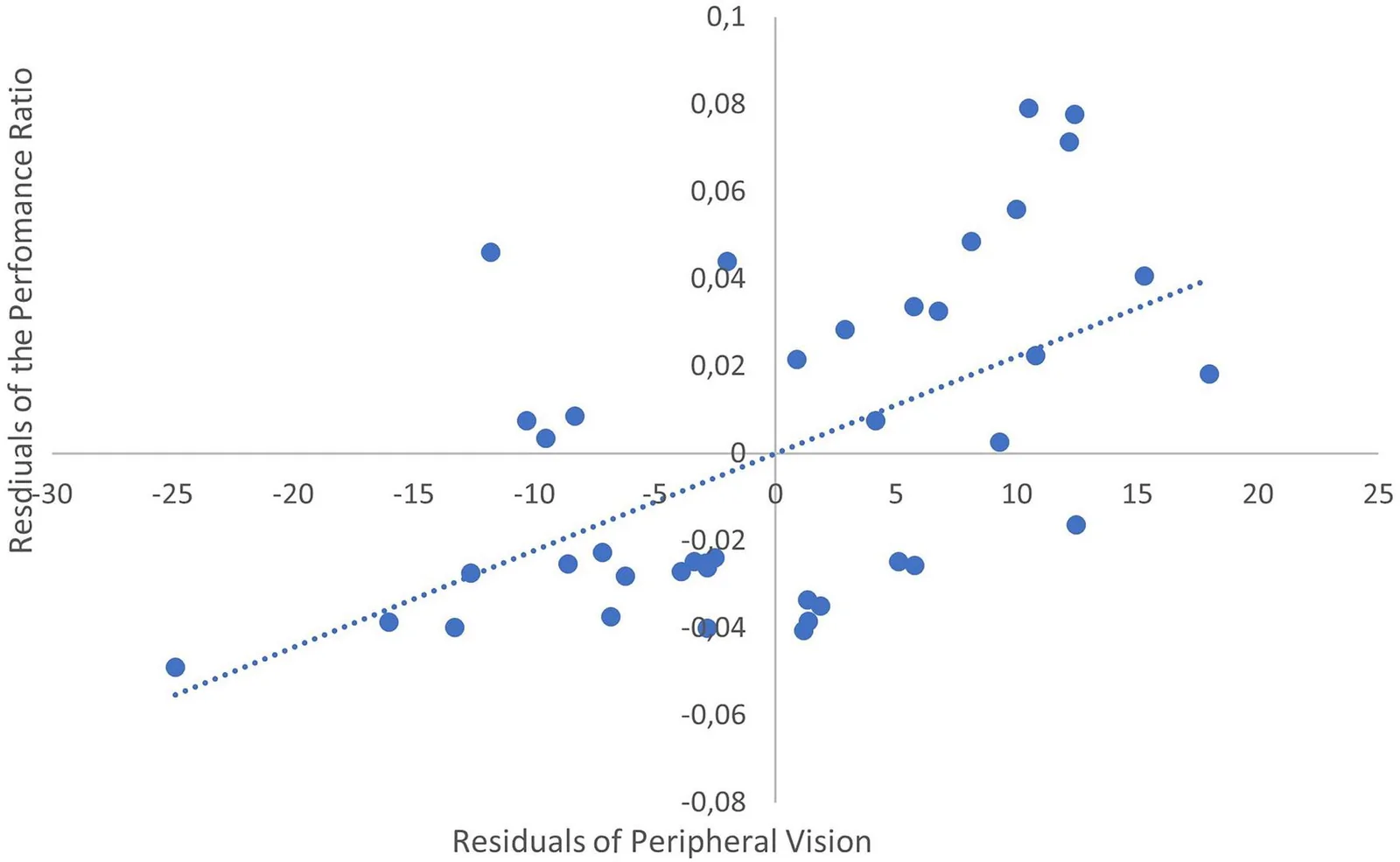
Partial regression plot (controlled for playing level) displaying the residuals of peripheral vision and residuals of the performance ratio.

To clarify the basis of the performance ratio, its underlying components (decision accuracy and decision time) were analyzed independently. Peripheral visual field extent showed a weak negative association with decision accuracy, r = −0.266, 95% CI [−0.534, 0.049], *p* = 0.048 (one-tailed; Figure 3). Given the small effect size and confidence interval including zero, this finding should be interpreted cautiously. The corresponding regression coefficient indicated that a 10° increase in peripheral visual field extent was associated with an estimated 1.8 percentage-point decrease in decision accuracy. In contrast, a stronger and statistically significant negative association was observed between peripheral visual field extent and decision time, r = −0.532, 95% CI [−0.724, −0.265], *p* < 0.001 (one-tailed; Figure 4), corresponding to an estimated 1273-ms reduction in decision time per 10° increase in peripheral visual field extent. Taken together, these results suggest that the association between peripheral vision and the performance ratio is more strongly reflected in decision time than in decision accuracy.

**FIGURE 3:**
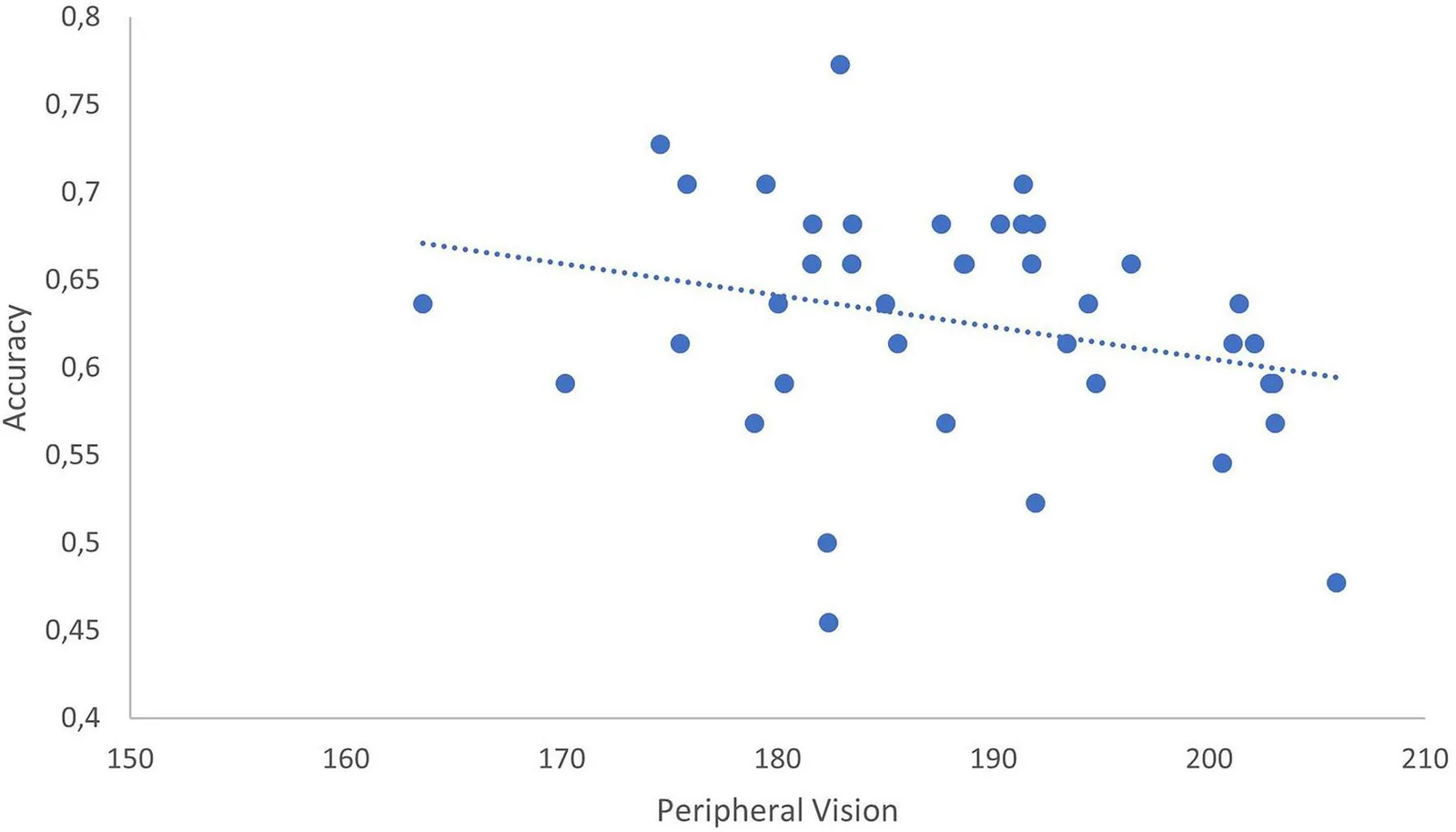
Relationship between peripheral vision and decision accuracy. The regression equation is y = - 0.0018x + 0.9668.

**FIGURE 4:**
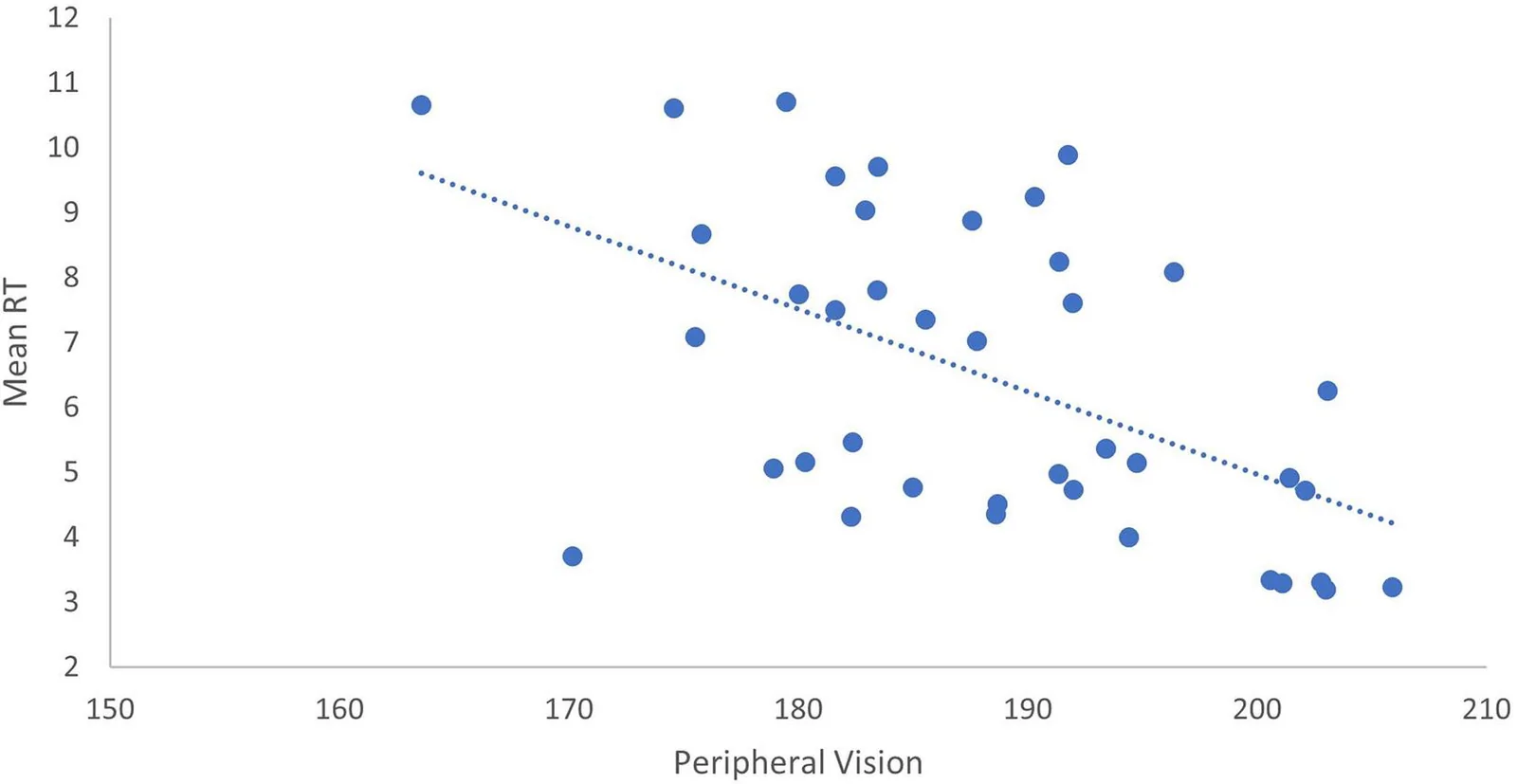
Relationship between peripheral vision and decision time. The regression equation is y = - 0.1273x + 30.437.

## 4 Discussion

The primary aim of the present study was to investigate whether individual differences in peripheral visual field extent are associated with decision-making efficiency under time pressure in soccer players, and whether this relationship persists after controlling for competitive level. The results were consistent with both hypotheses. Greater peripheral visual field extent was associated with greater decision-making efficiency as indexed by the performance ratio, and this relationship remained significant after controlling for players’ competitive level. This finding suggests that peripheral visual capabilities are related to decision-making efficiency beyond the influence of competitive experience. However, given that the performance ratio incorporates decision time as its denominator, this association should be interpreted cautiously. Analyses of the individual performance components indicated that the relationship was more strongly reflected in decision time than in decision accuracy.

These findings are consistent with theoretical accounts emphasizing the functional role of peripheral vision in supporting rapid extraction of spatial information in dynamic environments (Vater et al., 2016). In team sports such as soccer, where actions unfold under severe time constraints, the ability to extract relevant information without frequent gaze shifts may confer a performance advantage. Similar associations between visual field extent and performance have been reported in other domains, including visual search efficiency (Leachtenauer, 1978) and driving performance (Clay et al., 2005), suggesting that peripheral visual capacity may represent a more general perceptual resource relevant to goal-directed behavior in complex environments.

A more detailed analysis of the separate performance measures revealed that the relationship between peripheral vision and decision-making efficiency was more strongly reflected in decision time than in decision accuracy. Larger peripheral visual fields were moderately associated with faster responses, whereas the association with accuracy was weak. The observed association with decision time should therefore be interpreted alongside the weaker and statistically uncertain relationship with accuracy. The small effect size observed for accuracy (approximately 7% shared variance) suggests that differences in decision accuracy were less strongly associated with peripheral visual field extent than differences in decision time. However, the present study did not manipulate response instructions, and therefore cannot determine the mechanisms underlying this pattern. Future experimental studies incorporating different response instructions or explicit speed–accuracy manipulations are needed to investigate these mechanisms directly.

From a functional perspective, this speed advantage is plausible in dynamic sport environments. In soccer, decision windows are often extremely short, and delayed actions can reduce or eliminate tactical opportunities (Vater et al., 2016; Aksum et al., 2021). A larger functional visual field may allow players to monitor relevant off-ball information while maintaining central fixation, thereby reducing the need for time-consuming saccadic eye movements and supporting faster information uptake. This interpretation aligns with the concept of a “visual pivot,” whereby athletes stabilize their gaze on central task-relevant cues while extracting peripheral information to guide decisions (Vater et al., 2019).

Beyond the proposed visual pivot mechanism (Vater et al., 2019), neurophysiological properties of peripheral vision may also contribute to faster decision responses. The magnocellular pathway, which plays an important role in peripheral visual processing, exhibits higher temporal resolution compared with the parvocellular pathway (Livingstone and Hubel, 1987, 1988), potentially facilitating the rapid detection and processing of peripheral stimuli. In addition, visual field anisotropies indicate that motion sensitivity and temporal resolution vary across different regions of the visual field (Tarita-Nistor et al., 2008, 2014), which may contribute to inter-individual differences in peripheral visual processing speed.

The observed association between peripheral vision and decision accuracy was weak, indicating that differences in decision accuracy were less strongly associated with peripheral visual field extent than differences in decision time. Given the small magnitude of this association, accuracy differences appear to play a relatively minor role in explaining overall performance differences in the present task. However, as the present study did not manipulate response strategies, the mechanisms underlying this pattern remain uncertain and should be directly tested in future experimental designs.

It is important to emphasize the correlational nature of the present study. Although the association between peripheral visual field extent and decision-making efficiency remained significant after controlling for playing level, this finding does not establish that peripheral visual field extent independently contributes to or improves decision-making performance. It remains possible that unmeasured factors, such as general processing speed, attentional capacity (Piras, 2025), or sport-specific experience, influence both peripheral visual field extent and decision-making performance. Future research using longitudinal or experimental designs is needed to determine the causal mechanisms underlying this relationship and to investigate whether targeted peripheral vision training can enhance decision-making efficiency.

From an applied perspective, these findings suggest that peripheral visual field extent may be relevant for understanding individual differences in perceptual-cognitive performance in soccer. In positions characterized by high temporal constraints, such as wide attacking roles, faster perceptual processing may be particularly advantageous. In contrast, positions that require highly consequential decision-making in central areas may place relatively greater emphasis on accuracy. However, these positional interpretations remain speculative and should be directly tested in future position-specific studies.

The present findings may also have relevance for understanding how restrictions in the visual field affect elite performance under match conditions. Anecdotal reports from elite players and coaches during recent international tournaments have highlighted perceived impairments in visual perception when wearing protective facial equipment. While speculative, the present data suggest that even relatively small reductions in usable peripheral visual field could be associated with measurable changes in decision speed under time-constrained conditions. However, direct experimental evidence in real match settings is required to substantiate this claim.

Previous research suggests that aspects of peripheral vision and related perceptual skills may be modifiable through targeted training interventions (Graham et al., 2018). For example, improvements in peripheral detection and reaction speed have been reported following perceptual-motor training programs (Murata et al., 2023; Houston et al., 2016), as well as sport-specific visual training interventions (Piva et al., 2025). Murata et al. (2023) demonstrated that 12 weeks of Life Kinetik^®^ training significantly increased peripheral visual field extent and improved selective reaction time in high school soccer players compared with a control group. Similarly, Houston et al. (2016) reported that a 6-h computer-based perceptual-motor training program increased peripheral detection rates from 70% to 95% in individuals with visual field deficits, suggesting that even substantial limitations in peripheral vision may be amenable to targeted intervention. In a sports-specific context, Piva et al. (2025) found that an 8-week soccer-specific vision training program using the BlazePod system – the same apparatus employed in the present study to assess peripheral vision – significantly reduced visuomotor reaction time and improved saccadic precision in young soccer players. While these findings indicate potential for training-induced improvements, the extent to which such adaptations transfer to complex decision-making in competitive match play remains unclear and requires further investigation.

Several limitations should be acknowledged. First, peripheral visual field was assessed in a static measurement context, whereas in-game perception occurs under highly dynamic and interactive conditions. Second, decision-making was measured using video-based stimuli and verbal responses, which do not fully capture the motor and tactical constraints of real match play. Third, although competitive level was statistically controlled, it remains a coarse proxy for expertise and may not capture all relevant dimensions of skill. Future research should integrate eye-tracking methodologies with peripheral vision assessment in more ecologically valid environments, potentially including immersive virtual reality systems once their field-of-view limitations have been resolved.

In conclusion, the present study provides evidence that individual differences in peripheral visual field extent are positively associated with decision-making efficiency in soccer players, even after controlling for competitive level. This relationship is more strongly reflected in faster decision times than in accuracy differences. However, the correlational nature of the study does not allow conclusions about the mechanisms underlying this pattern or about participants’ response strategies. Taken together, these findings extend previous research on visual perception in dynamic environments by demonstrating an association between peripheral visual field extent and individual differences in perceptual-cognitive performance in sport.

## Data Availability

The raw data supporting the conclusions of this article will be made available by the authors, without undue reservation.

## References

[B1] Aksum K. M. Magnaguagno L. Bjørndal C. T. Jordet G. (2021). Scanning activity of elite football players in 11 vs. 11 match play: An eye-tracking analysis on the duration and visual information of scanning. *PLoS One* 16:e0244118. 10.1371/journal.pone.0244118 34415909 PMC8378692

[B2] BlazePod (2018). *BlazePod (Version 5.5.1) [Mobile app].* Cupertino, CA: App Store.

[B3] Clay O. J. Wadley V. G. Edwards J. D. Roth D. L. Roenker D. L. Ball K. K. (2005). Cumulative meta-analysis of the relationship between useful field of view and driving performance in older adults: Current and future implications. *Optometry Vis. Sci.* 82, 724–731. 10.1097/01.opx.0000175009.08626.65 16127338

[B4] Cohen J. (1988). *Statistical Power Analysis for the Behavioral Sciences*, 2nd Edn. Mahwah, NJ: Lawrence Erlbaum Associates.

[B5] DeCouto B. S. Fawver B. Thomas J. L. Williams A. M. Vater C. (2023). The role of peripheral vision during decision-making in dynamic viewing sequences. *J. Sports Sci.* 41, 1852–1867. 10.1080/02640414.2023.2301143 38234241

[B6] de-Oliveira L. A. Matos M. V. Fernandes I. G. Nascimento D. A. da Silva-Grigoletto M. E. (2021). Test-retest reliability of a visual-cognitive technology (BlazePod™) to measure response time. *J. Sports Sci. Med.* 20, 179–180. 10.52082/jssm.2021.179 33708001 PMC7919356

[B7] Erdfelder E. Faul F. Buchner A. (1996). GPOWER: A general power analysis program. *Behav. Res. Methods Instruments Comp.* 28, 1–11. 10.3758/BF03203630

[B8] Graham V. Napier Dovorany K. Bruton E. Richards N. Garbus J. Garbus C. (2018). The effect of relaxation techniques and visual training on peripheral vision in U.S. collegiate soccer players. *Optom. Vis. Perform.* 6, 79–86.

[B9] Heijl A. Patella V. M. Bengtsson B. (1989). *The Field Analyzer Primer.* Germany: Carl Zeiss Meditec.

[B10] Houston K. E. Bowers A. R. Fu X. Liu R. Goldstein R. B. Churchill J. et al. (2016). A pilot study of perceptual-motor training for peripheral prisms. *Transl. Vis. Sci. Technol.* 5:9. 10.1167/tvst.5.1.9 26933522 PMC4771076

[B11] Hüttermann S. Memmert D. (2017). The attention window: A narrative review of limitations and opportunities influencing the focus of attention. *Res. Quar. Exerc. Sport* 88, 169–183. 10.1080/02701367.2017.1293228 28332919

[B12] Klostermann A. Vater C. Kredel R. Hossner E.-J. (2020). Perception and action in sports: On the functionality of foveal and peripheral vision. *Front. Sports Act. Liv.* 1:66. 10.3389/fspor.2019.00066 33344989 PMC7739830

[B13] Leachtenauer J. C. (1978). Peripheral acuity and visual search performance. *Proc. Hum. Fact. Soc. Ann. Meet.* 22, 199–203. 10.1177/001872087802000504

[B14] Livingstone M. S. Hubel D. H. (1987). Psychophysical evidence for separate channels for the perception of form, color, movement, and depth. *J. Neurosci.* 7, 3416–3468. 10.1523/JNEUROSCI.07-11-03416.1987 3316524 PMC6569044

[B15] Livingstone M. S. Hubel D. H. (1988). Segregation of form, color, movement, and depth: Anatomy, physiology, and perception. *Science* 240, 740–749. 10.1126/science.3283936 3283936

[B16] Mann D. T. Williams A. M. Ward P. Janelle C. M. (2007). Perceptual-cognitive expertise in sport: A meta-analysis. *J. Sport Exerc. Psychol.* 29, 457–478. 10.1123/jsep.29.4.457 17968048

[B17] Mizusawa K. Sweeting R. L. Knouse S. B. (1983). Comparative studies of color fields, visual acuity fields, and movement perception limits among varsity athletes and non-varsity groups. *Percept. Motor Skills* 56, 887–892. 10.2466/pms.1983.56.3.887 6877976

[B18] Murata Y. Wachi M. Jiromaru T. Onishi H. Fujitani R. Noguchi S. et al. (2023). The effect of Life Kinetik^®^ training on visual function. *Rigakuryoho Kagaku* 38, 289–293. 10.1589/rika.38.289

[B19] Panchuk D. Maloney M. (2022). A perception-action assessment of the functionality of peripheral vision in expert and novice Australian footballers. *J. Sport Exerc. Psychol.* 44, 327–334. 10.1123/jsep.2021-0121 35894962

[B20] Piras A. (2025). The role of the peripheral target in stimulating eye movements. *Psychol. Sport Exerc.* 76:102744. 10.1016/j.psychsport.2024.102744 39307329

[B21] Piva T. Zerbini V. Barducco A. Mancioppi S. Raisi A. Vassali M. et al. (2025). Reduction in reaction time and improved oculomotor function following football-specific vision training in young players. *BMC Sports Sci. Med. Rehabil.* 17:298. 10.1186/s13102-025-01368-z 41102801 PMC12532858

[B22] Savelsbergh G. J. P. van der Kamp J. Williams A. M. Ward P. (2005). Anticipation and visual search behaviour in expert soccer goalkeepers. *Ergonomics* 48, 1686–1697. 10.1080/00140130500101346 16338733

[B23] Spielman R. M. Jenkins W. J. Lovett M. D. (2020). *Psychology 2e.* Houston, TX: OpenStax, Rice University.

[B24] Stankov A. D. Touryan J. Gordon S. Ries A. J. Ki J. Parra L. C. (2021). During natural viewing, neural processing of visual targets continues throughout saccades. *J. Vis.* 21:7. 10.1167/jov.21.10.7 PMC8431980 34491271

[B25] Stark L. Bridgeman B. (1983). Role of corollary discharge in space constancy. *Percept. Psychophys.* 34, 371–380. 10.3758/BF03203050 6657439

[B26] Tarita-Nistor L. González E. G. Markowitz S. N. Lillakas L. Steinbach M. J. (2008). Increased role of peripheral vision in self-inducedmotion in patients with age-related macular degeneration. *Invest. Ophthalmol. Vis. Sci.* 49, 3253–3258. 10.1167/iovs.07-1290 18390642

[B27] Tarita-Nistor L. Hadavi S. Steinbach M. J. Markowitz S. N. González E. G. (2014). Vection in patients with glaucoma. *Optometry Vis. Sci.* 91, 556–563. 10.1097/OPX.0000000000000233 24681830

[B28] Urry K. Burns N. R. Baetu I. (2015). Accuracy-based measures provide a better measure of sequence learning than reaction time-based measures. *Front. Psychol.* 6:1158. 10.3389/fpsyg.2015.01158 26321982 PMC4531304

[B29] Vandierendonck A. (2017). A comparison of methods to combine speed and accuracy measures of performance: A rejoinder on the binning procedure. *Behav. Res. Methods* 49, 653–673. 10.3758/s13428-016-0721-5 26944576

[B30] Vater C. Kredel R. Hossner E.-J. (2016). Detecting single-target changes in multiple object tracking: The case of peripheral vision. *Attent. Percept. Psychophys.* 78, 1004–1019. 10.3758/s13414-016-1078-7 26942770

[B31] Vater C. Pinchuk S. Vukojević B. (2026). Soccer players’ ability to use peripheral vision is affected by viewing angles but not crowding. *Curr. Issues Sport Sci.* 11:040. 10.36950/2026.2ciss040

[B32] Vater C. Williams A. M. Hossner E. J. (2019). What do we see out of the corner of our eye? The role of visual pivots and gaze anchors in sport. *Intern. Rev. Sport Exerc. Psychol.* 13, 81–103. 10.1080/1750984X.2019.1582082

[B33] Vickers J. N. (2007). *Perception, Cognition, and Decision Training: The Quiet Eye in Action.* Champaign, Ill: Human Kinetics.

[B34] Walker H. K. Hall W. D. Hurst J. W. (Eds.). (1990). *Clinical Methods: The History, Physical, and Laboratory Examinations*, 3rd Edn. Oxford: Butterworths. 21250045

[B35] Williams A. M. Ford P. R. (2008). Expertise and expert performance in sport. *Intern. Rev. Sport Exerc. Psychol.* 1, 4–18. 10.1080/17509840701836867

[B36] Williams A. M. Ford P. R. Eccles D. W. Ward P. (2011). Perceptual-cognitive expertise in sport and its acquisition: Implications for applied psychology. *Appl. Psychol. An Intern. Rev.* 25, 432–442. 10.1002/acp.1710

[B37] Wozniak K. (2019). *U19-Bundesliga ist kein Freifahrtschein.* Germany: RevierSport.

[B38] Wu F. Li K. Dou W. Wang C. Zhang Z. (2025). How does peripheral vision affect driver performance when passing on roads with lateral traffic conflicts? A simulated driving experiment. *Intern. J. Indust. Ergon.* 110:103134. 10.1016/j.ergon.2025.103828

